# Clinical effectiveness of mupirocin for preventing S. aureus infections in non-surgical settings: a meta-analysis

**DOI:** 10.1186/2047-2994-4-S1-O5

**Published:** 2015-06-16

**Authors:** R Nair, E Perencevich, A Blevins, M Goto, R Nelson, ML Schweizer

**Affiliations:** 1 Epidemiology, University of Iowa, Iowa City, IA, USA; 2 Internal Medicine, University of Iowa, Iowa City, IA, USA; 3 Library Sciences, University of Iowa, Iowa City, IA, USA; 4 Internal Medicine, University of Utah, Salt Lake City, UT, USA

## Introduction

A protective effect of mupirocin has been seen among surgical, nonsurgical and dialysis patients. Our aim is to summarize evidence for mupirocin decolonization for prevention of *S. aureus* infections in non-surgical healthcare settings.

## Objectives

To identify the optimal setting and patient population to implement mupirocin decolonization for prevention of *S. aureus* infections using meta-analytic methods.

## Methods

We conducted systematic searches in PubMed, Cochrane Library Databases, Scopus, Web of Science, and ClinicalTrials.gov to identify papers published until 2013 on effectiveness of mupirocin in healthcare settings. Two investigators independently abstracted data with a pilot-tested form. Risk of bias was assessed using the Cochrane tool. The crude odds ratios were pooled (cpOR) using a random-effects model. Heterogeneity was evaluated using the Woolf’s test for homogeneity and *I^2^* statistics.

## Results

Of the 12,644 studies identified, 8 randomized controlled trials and 19 quasi-experimental studies met the study inclusion criteria. Mupirocin was observed to reduce the odds of *S. aureus* infections by 70% (cpOR=0.30, 95% CI 0.23, 0.39) and 60% (cpOR=0.40, 95% CI 0.27, 0.62) in both dialysis and non-dialysis settings, respectively. Nevertheless, there was highly significant (p=0.0009) and moderate heterogeneity (*I^2^*=46%) among studies. Studies were homogeneous (p>0.1) when stratified analyses were performed by specific clinical settings. Among the 6 studies that took place in adult intensive care units (ICUs), mupirocin decolonization was associated with a 56% reduction in the odds of *S. aureus* infection (cpOR=0.44, 95% CI 0.26, 0.73). There was also a protective effect of mupirocin against *S. aureus* exit site infections among patients undergoing peritoneal dialysis (cpOR=0.23, 95% CI 0.15, 0.36) and against bacteremia among hemodialysis patients (cpOR=0.15, 95%CI 0.06, 0.36).

## Conclusion

Mupirocin decolonization is protective against *S. aureus* infections among both dialysis and adult ICU patient populations. Future studies should target other patient settings such as long-term care facilities.

## Disclosure of interest

None declared.

